# Changes in Distance Ocular Deviation After Smartphone Viewing in Young Adults Without Manifest Strabismus

**DOI:** 10.3390/jemr19040086

**Published:** 2026-08-12

**Authors:** Miharu Mihara, Noriko Katayama, Ken Kakeue, Mitsuya Otsuka, Kazuichi Maruyama, Atsushi Hayashi

**Affiliations:** Department of Ophthalmology, Graduate School of Medicine and Pharmaceutical Sciences, University of Toyama, Toyama 930-0194, Japan

**Keywords:** smartphone use, ocular deviation, exophoria, gaze movement, vergence, eye tracking

## Abstract

Background: This prospective observational study assessed acute changes in distance ocular deviation after brief smartphone viewing in young adults without manifest strabismus and identified factors associated with the magnitude of change. Methods: Fifty-nine healthy young adults without strabismus (aged 20–30 years; mean ± standard deviation, 23.2 ± 2.5 years) wore a head-mounted eye-tracking system and viewed a smartphone for 30 min. Ocular deviation angles at near and distance fixation were measured immediately before and after smartphone viewing. During smartphone viewing, eye-movement parameters and smartphone distance were continuously recorded. Results: Before smartphone viewing, the mean deviation angles were −6.2 ± 6.8^Δ^ and −2.0 ± 2.8^Δ^ at near and distance fixation, respectively (negative values indicate exodeviation). After smartphone viewing, deviation angles shifted toward less exodeviation, measuring −4.3 ± 6.6^Δ^ and −1.1 ± 2.4^Δ^ at near and distance fixation, respectively. The magnitude of exodeviation reduction was negatively correlated with baseline ocular deviation, indicating that individuals with larger pre-viewing exodeviation exhibited greater alignment shifts after smartphone viewing. Eye-movement analysis revealed that gaze movements of <30°/s accounted for 57.8% of detected gaze-movement events. Conclusions: Thirty minutes of smartphone viewing was followed by a reduction in exodeviation at near and distance fixation in young adults without manifest strabismus. Greater baseline exodeviation was associated with a larger reduction, and low-velocity gaze movements predominated.

## 1. Introduction

Recently, smartphone ownership has increased globally, with an estimated 5.6 billion users worldwide [1], particularly among young people and children. According to Japanese government statistics, 46.2% of elementary school students aged ≥10 years, 82% of junior high school students, and 97.6% of high school students use smartphones to access the internet [2].

Since the publication of Lee et al.’s report [3], case series and observational studies have increasingly described acute acquired concomitant esotropia (AACE) in young individuals associated with excessive smartphone and digital device use [4,5,6,7,8]. This condition raises two major concerns: first, many affected individuals develop esotropia despite having no prior history of strabismus or amblyopia in childhood and having maintained good near stereopsis; second, reducing smartphone use alone does not lead to full recovery in many cases, and strabismus surgery is often required. Although early detection may increase the likelihood of improvement through reduced digital device use [5], most patients aged 13–35 years wait more than 6 months after the onset of distant diplopia—the first symptom of AACE—before seeking ophthalmologic care [6]. These findings indicate that prolonged, excessive, and close-range smartphone use may trigger AACE, emphasizing the importance of prevention.

Furthermore, prolonged smartphone viewing has been associated with symptoms of digital eye strain, including visual fatigue, blurred vision, and binocular vision complaints [9,10,11]. Recent reviews have suggested that digital eye strain involves accommodative and vergence dysfunction in addition to ocular surface abnormalities, while emphasizing that objective assessments of binocular visual function remain limited [10]. These observations highlight the need for the objective evaluation of ocular alignment during smartphone viewing.

Several studies have examined the effects of smartphone use on accommodative lag, accommodative function, and fusional convergence in individuals without strabismus [12,13,14]. However, early functional changes in ocular alignment that may precede the clinical onset of AACE remain poorly understood. In particular, it is unclear whether short-term smartphone viewing can induce measurable alterations in distance ocular alignment in non-strabismic individuals before overt esotropia develops. Therefore, this study aimed to investigate changes in ocular deviation before and after smartphone use in healthy young adults without strabismus and to identify factors associated with these changes, with a particular focus on distance ocular alignment.

## 2. Materials and Methods

This study was approved by the Ethics Committee of the University of Toyama (approval #: R2024070) and adhered to all local laws and principles outlined in the Declaration of Helsinki. Written informed consent was obtained from each participant after the experimental procedure was fully explained.

### 2.1. Participants

Sixty healthy young adults without manifest strabismus on cover–uncover tests for both distance and near fixation participated in this study. Participants were aged 18–30 years and had a best-corrected visual acuity of logarithm of the minimum angle of resolution (logMAR) of 0.0 or better in each eye, as well as stereo acuity ≤ 60 arc seconds. Participants with a history of strabismus, amblyopia, other ocular diseases except refractive error, or neurological or systemic diseases related to eye-movement disorders were excluded. In addition, during this study, participants who exhibited manifest strabismus at least once in each cover–uncover test were excluded.

### 2.2. Ophthalmologic Examination

**Figure 1:**
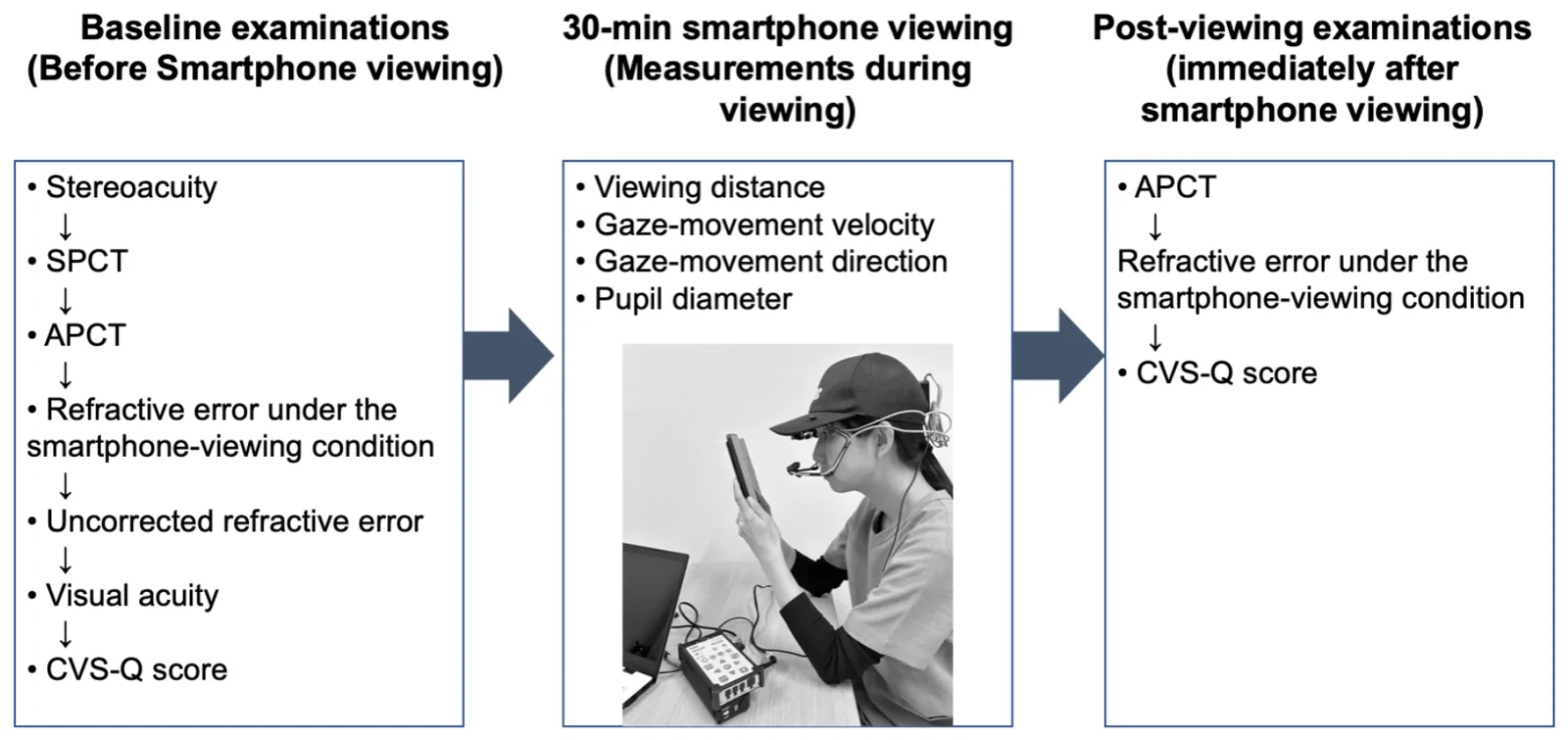
Study flow diagram. Participants underwent baseline ophthalmic examinations before smartphone viewing, continuous measurements during 30 min of unrestricted smartphone viewing, and repeat ophthalmic examinations immediately after smartphone viewing. APCT: alternate prism cover test; CVS-Q: Computer Vision Syndrome Questionnaire; SPCT: single-prism cover test.

All participants underwent measurements of visual acuity, uncorrected refractive error, residual refractive error with habitual correction (reflecting refractive status while viewing the smartphone with usual correction), ocular deviation angle, and stereopsis before smartphone viewing (Figure 1). Refractive error was measured using the Handy Ref-K autorefractor (Nidek Co., Ltd., Gamagori, Japan), while near and distance stereo acuity was evaluated using the Titmus Fly Test and the System Chart SC-1600 Pola (Nidek Co., Ltd., Gamagori, Japan). Angles of ocular deviation at near (30 cm) and distance (5 m) fixation were measured using the alternate prism cover test (APCT) and single-prism cover test (SPCT). Furthermore, residual refractive error with habitual correction and ocular deviation angle were measured immediately after smartphone use. Ocular deviation was measured under habitual refractive correction. Participants who habitually wore soft contact lenses were examined with their contact lenses in place, whereas habitual spectacle wearers were examined while wearing their spectacles. The same correction was used for the pre- and post-viewing measurements. Ophthalmic examinations before smartphone use were performed on the same day in the following order: stereoscopic vision test, eye position test, refraction test, and visual acuity test. Ophthalmic examinations after smartphone use (Figure 1) were performed by different examiners who were unaware of the pre-use results; all examinations were completed within 5 min after the completion of smartphone use.

### 2.3. Examination Paradigm

Participants used handheld smartphones for 30 min while seated, at a self-selected optimal viewing distance. Participants viewed the smartphone while wearing an eye tracker (EMR-9; nac Image Technology Inc., Tokyo, Japan) and the EyeCare Clip (Clearelectron Co., Ltd., Tokyo, Japan) (Figure 1). The ambient room illuminance during smartphone use was 351–416 lux (mean ± SD, 364 ± 10 lux). Participants used their own smartphones with their usual screen brightness settings, which were not standardized by the investigators. Smartphone screen sizes ranged from 4.7 to 6.3 inches. Smartphones were held in portrait orientation, and only social networking, video viewing, and web browsing were permitted; gaming and vertically oriented e-books were prohibited. All participants could clearly view the smartphone either with uncorrected vision or soft contact lenses, depending on their refractive error. Participants completed the Computer Vision Syndrome Questionnaire (CVS-Q) [15] before and immediately after smartphone use.

### 2.4. Eye-Movement Recording

During smartphone use, eye position and pupil size were recorded using the EMR-9 at a sampling rate of 240 Hz. The eye tracker recorded participants’ eye positions using the dark pupil technique. Before testing, a 9-point grid (3 × 3 matrix) calibration and subsequent validation procedures were performed for each participant using the software supplied by the eye tracker manufacturer. The smartphone viewing distance was measured using the EyeCare Clip, but due to the characteristics of its measurement principle and the small screen size of smartphones, there is a risk of overestimating the viewing distance [16]. Therefore, to prevent viewing distance overestimation, the smartphone was pre-attached to the left edge of a plastic board (14.8 cm × 21.0 cm) at the angle of the subject’s gaze when looking at the smartphone.

### 2.5. Data Analysis and Statistics

Data recorded with the EMR-9 were analyzed offline using EMR-dFactory software (Ver. 2.72; nac Image Technology Inc., Tokyo, Japan). Gaze-movement velocity, direction, and pupil diameter during smartphone viewing were calculated.

Continuous variables are presented as mean ± standard deviation (SD) and categorical variables as counts and percentages. The Wilcoxon signed-rank test compared residual refractive error (spherical equivalent [SE]), ocular deviation angle, and CVS-Q score before and after viewing.

To examine factors independently associated with changes in ocular deviation, multivariable linear regression analysis was performed. Previous studies have reported that patients with AACE most commonly present with distance diplopia as the initial symptom [17]. Therefore, the dependent variable was the change in ocular deviation at distance fixation (changes in the APCT at distance). Independent variables included baseline SE, interocular SE difference, APCT at distance before smartphone viewing, and viewing distance during smartphone viewing, which were selected based on their clinical relevance. Multicollinearity was assessed using the variance inflation factor (VIF), where variables with VIF > 5 were considered to indicate significant collinearity. Regression coefficients (β) with 95% confidence intervals (CIs) were calculated. Statistical analyses were performed using JMP Pro software (version 18.0.1; SAS Institute, Cary, NC, USA) and R software (version 4.3.1; R Foundation for Statistical Computing, Vienna, Austria), and a two-sided *p*-value < 0.05 was considered statistically significant.

## 3. Results

### 3.1. Participants

Fifty-nine of sixty participants were included in the study (mean age, 23.2 ± 2.5 years; range, 20–30 years) (Table 1), as one participant was excluded because intermittent exotropia was detected during the study on the cover–uncover test. Thirty-five participants were women, and thirty-two used smartphones while wearing soft contact lenses for refractive correction. No participant exhibited manifest strabismus on the cover–uncover test.

### 3.2. Pupil Size and Viewing Distance

The mean viewing distance to the smartphone was 24.7 ± 4.0 cm (range, 17.3 to 35.8 cm) (Supplementary Figure S1), and fifty-two participants viewed at <30 cm. Viewing distance data could not be obtained for one participant. The mean pupil diameter during viewing was 2.7 ± 0.7 mm (range, 1.6 to 5.4 mm) in the left eye and 2.7 ± 0.8 mm (range, 1.7 to 5.4 mm) in the right eye.

### 3.3. Ocular Deviation

**Figure 2:**
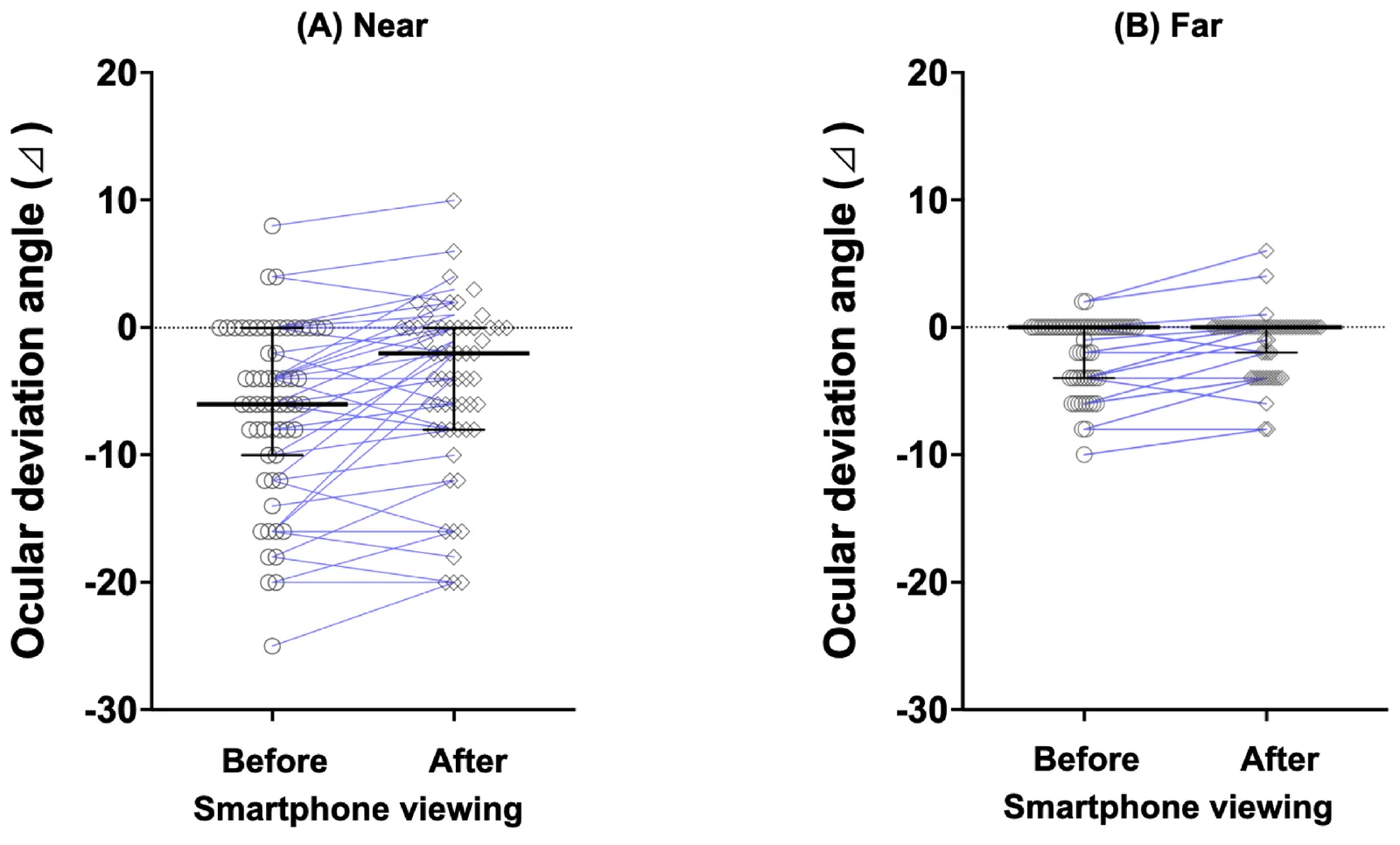
Individual ocular deviation measured by the alternate prism cover test at (**A**) near and (**B**) distance fixation before and after smartphone viewing. Negative values indicate exodeviation. Circles indicate values before smartphone viewing, and diamonds indicate values after smartphone viewing. Each line represents one participant; horizontal bars indicate the median; and error bars represent the interquartile range (IQR). Δ, prism diopters.

The ocular deviations measured by the APCT at near and distance fixation were −6.2 ± 6.8^Δ^ and −2.0 ± 2.8^Δ^, respectively, before smartphone viewing (negative values indicate exodeviation) (Table 1, Supplementary Figure S2A,B). At distance fixation, three participants had esophoria (2^Δ^), 26 had exophoria (−10^Δ^ to −1^Δ^), and 30 had orthophoria (no deviation). After smartphone viewing, deviation angles significantly decreased to −4.3 ± 6.6^Δ^ and −1.1 ± 2.4^Δ^ at near and distance fixation, respectively (*p* < 0.05 for both) (Figure 2A,B). The mean changes and standardized effect sizes for the pre–post differences are summarized in Supplementary Table S1. Individual responses varied for both APCT at near and distance fixation. At distance fixation, 35 participants (59.3%) showed no change, 22 (37.3%) exhibited an eso-directional shift (1–4^Δ^), and only 2 (3.4%) showed a small exo-directional shift (2^Δ^). At near fixation, 14 participants (23.7%) showed no change, 33 (55.9%) exhibited an eso-directional shift (1–14^Δ^), and 12 (20.3%) showed an exo-directional shift (2–4^Δ^).

### 3.4. Multivariable Linear Regression Analysis

Because AACE’s association with excessive smartphone use first manifests as distance diplopia due to a shift toward esotropia at distance fixation [16], we examined factors associated with the change in APCT at distance fixation (positive values indicate an eso-directional shift, i.e., a decrease in exodeviation or an increase in esodeviation) following smartphone viewing. Clinically significant factors and those with *p* < 0.2 in univariate analysis (Supplementary Table S2) were selected for inclusion in the multivariable model.

Variables meeting the inclusion criteria were entered into the multivariable linear regression model (Table 2). Baseline APCT at distance fixation was the only significant predictor of the reduction in exodeviation after smartphone viewing (β = −0.251, *p* < 0.001), indicating that greater baseline exodeviation was associated with a larger eso-directional shift. In contrast, viewing distance and interocular uncorrected SE differences were not significant contributors. No multicollinearity was observed (VIF < 2). To address the potential influence of mathematical coupling associated with the change-score analysis, we also performed a sensitivity analysis using post-viewing APCT-assessed distance deviation as the dependent variable while retaining the same explanatory variables as those in the primary multivariable model. Baseline distance APCT remained the only significant predictor (B = 0.749; 95% CI, 0.626–0.872; *p* < 0.001), whereas uncorrected SE in the right eye and interocular uncorrected SE difference were not significantly associated with post-viewing APCT-assessed distance deviation (Supplementary Table S3).

### 3.5. Gaze Movements

**Figure 3:**
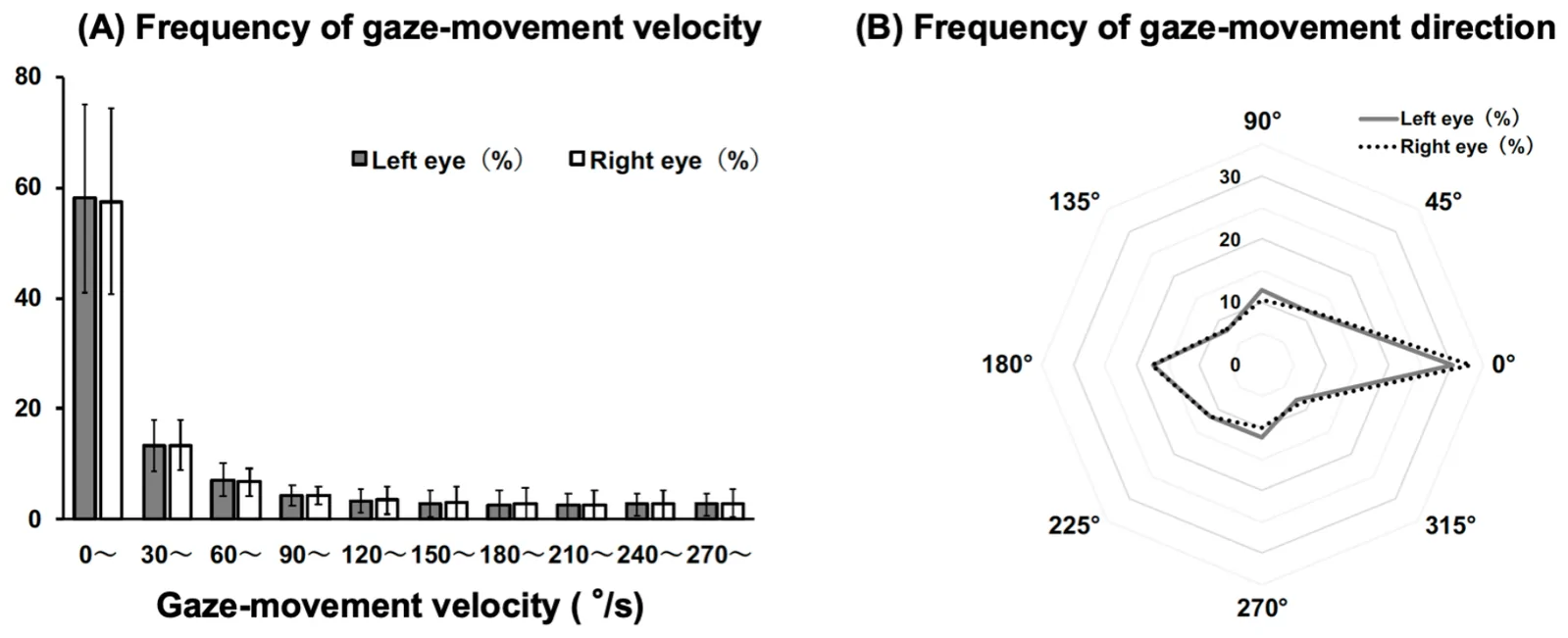
Characteristics of gaze movements during smartphone viewing. Frequency distribution of gaze-movement (**A**) velocities categorized into velocity bins (bars represent the percentage of gaze-movement events in each velocity bin for the left and right eyes) and (**B**) directions expressed in polar coordinates (solid line: left eye; dotted line: right eye).

During smartphone viewing, gaze-movement events with velocities of <30°/s accounted for 57.8% of all detected gaze-movement events (Figure 3A). Their frequency decreased progressively with increasing velocity, with gaze-movement events of ≥210°/s accounting for 8.0% of the total. The mean of the horizontal-to-vertical gaze-movement ratio was approximately 2:1 (Figure 3B). To explore whether these gaze-movement characteristics were associated with ocular alignment changes, we performed univariable analyses using the percentage of gaze-movement events with velocities of <30°/s and the horizontal-to-vertical gaze-movement ratio. Neither parameter was significantly associated with the change in distance APCT (Supplementary Table S2).

### 3.6. Refraction

The mean SE at baseline was −3.36 ± 2.25 D (range, −8.88 to +0.38 D) in the left eye and −3.57 ± 2.4 D (range, −8.88 to +0.25 D) in the right eye (Supplementary Figure S3A,B). The mean SE under habitual smartphone viewing correction (soft contact lenses or unaided) was −1.28 ± 1.58 D (range, −6.38 to +0.38 D) in the left eye and −1.31 ± 1.66 D (range, −7.13 to +0.25 D) in the right eye (Supplementary Figure S3C,D). After smartphone viewing, these values were −1.42 ± 1.63 D (range, −7.38 to +0.75 D) in the left eye and −1.41 ± 1.63 D (range, −7.25 to +0.25 D) in the right eye. Although the SE shifted slightly towards myopia before and after smartphone use, the change was not significant (left eye, *p* = 0.07; right eye, *p* = 0.15).

### 3.7. CVS-Q Questionnaire

The CVS-Q score before and after smartphone use was 2.1 ± 2.5 and 2.4 ± 2.2, respectively. There was no significant change in the CVS-Q scores before and after smartphone use (*p* = 0.33). Regarding subjective symptoms immediately after smartphone use, two experienced diplopia at distance fixation, four experienced blurred vision at a distance fixation, and seven experienced blurred vision at a near distance fixation. However, in all cases, the symptoms improved within 30 min to the next day.

## 4. Discussion

The principal finding of this study was a significant shift toward less exodeviation after 30 min of smartphone viewing, which was observed at both near and distance fixation. Importantly, multivariable analysis indicated that the magnitude of the shift toward convergence (reduction in exodeviation) was driven primarily by baseline ocular alignment—particularly baseline distance APCT—whereas viewing distance and refractive parameters were not significant contributors. Eye-tracking analysis demonstrated that low-velocity gaze movements accounted for a substantial proportion of viewing time, suggesting limited scanning behavior during smartphone viewing.

Ocular alignment showed a significant reduction in exophoric deviation following smartphone use, indicating an eso-directional shift. Although none of the participants had manifest strabismus on cover–uncover testing, orthophoria and slight exodeviation on APCT were common at baseline. The strong association between the baseline distance APCT and post-viewing eso-directional shift suggests that baseline ocular alignment is associated with the response of ocular alignment to brief smartphone near-work viewing. Collectively, these findings suggest that sustained near viewing can bias vergence control such that ocular alignment does not immediately return to its pre-viewing state when fixation shifts back to distance, particularly in individuals with specific baseline alignment profiles. However, because the outcome variable was defined as the change from baseline, this association should be interpreted with caution, as mathematical coupling and regression to the mean may have contributed to the observed association. Nevertheless, an additional sensitivity analysis using post-viewing APCT-assessed distance deviation as the dependent variable yielded consistent findings, with baseline distance APCT remaining the only significant predictor.

Although closer viewing distances generally increase convergence demand [18], viewing distance did not emerge as a significant predictor in the multivariable model. This may be explained by the limited variability within this cohort: approximately 90% of participants viewed their smartphones from <30 cm, which could reduce the ability to detect distance-dependent effects. It is also plausible that task characteristics (e.g., sustained fixation with limited gaze shifts) contribute to alignment changes beyond viewing distance alone.

Allen and Mehta conducted a pilot study investigating the effects of 30 min of habitual smartphone use on accommodative and convergence functions in healthy young adults and reported no statistically significant changes in near point of accommodation, near point of convergence, or accommodative facility after correction for multiple comparisons [14]. Similar to their study, participants in the present study used smartphones for 30 min, but the outcome measures differed: while Allen and Mehta focused on conventional functional measures of the near triad [14], the present study quantified pre–post changes in ocular alignment and refractive status. This difference in endpoints may partly account for the apparent discrepancy: subtle alignment shifts may occur even in the absence of detectable changes in traditional near-triad measures, particularly after sustained smartphone near work. Thus, our findings do not necessarily contradict previous reports but instead suggest that ocular alignment may be a more sensitive indicator of the immediate effects of sustained smartphone viewing than conventional near-triad measures. However, the present findings do not provide sufficient evidence to conclude that these alignment changes represent an early functional alteration preceding AACE or are specific to smartphone viewing.

The relatively small mean change partly reflects the fact that many participants showed little or no change in ocular alignment, whereas a substantial proportion exhibited small but measurable eso-directional shifts. However, because the observed change in ocular deviation was modest, the inherent measurement variability of APCT should be considered when interpreting the findings. Previous studies have demonstrated good repeatability of APCT in experienced examiners when measuring horizontal phorias in healthy adults, although a certain degree of measurement variability is unavoidable [19]. Studies of patients with strabismus have similarly highlighted the importance of considering measurement variability when interpreting repeated APCT measurements [20,21]. Although post-viewing examiners were masked to baseline measurements, a possible inter-examiner effect cannot be completely excluded. Notably, however, the magnitude of ocular alignment change was significantly associated with baseline distance exodeviation, suggesting that the observed pattern is unlikely to be explained solely by measurement variability. In addition, the standardized effect sizes for the observed pre–post changes were 0.50 and 0.60 at near and distance fixation, respectively, indicating that the observed changes were not negligible despite their modest absolute magnitudes. Nevertheless, the relatively small change in mean warrants cautious interpretation.

Eye-movement analysis revealed that more than half of the gaze movements had velocities < 30°/s. The small screen size and frequent scrolling likely reduce the need for rapid, large-amplitude gaze shifts. Across participants, the mean horizontal-to-vertical gaze-movement ratio was approximately 2:1. This predominance of horizontal gaze movements may partly reflect visual characteristics of some smartphone tasks, such as web browsing or text-based content, whereas vertical content displacement may be achieved largely through scrolling. Because viewing content was not standardized, different visual tasks may also have influenced gaze-movement patterns. Neither the proportion of gaze-movement events with velocities of <30°/s nor the horizontal-to-vertical gaze-movement ratio was significantly associated with the magnitude of change in distance APCT. Therefore, these findings provide a descriptive characterization of gaze behavior during smartphone viewing; however, their contribution to the observed ocular alignment changes remains unclear.

In contrast to ocular alignment, objective refraction did not change significantly immediately after smartphone viewing. This is consistent with prior reports indicating that near-work-induced transient myopic shifts associated with smartphone reading may be small (approximately 0.13 D) [22]. Because all participants wore eye trackers, habitual spectacle wearers viewed the smartphone without their glasses, whereas contact lens users viewed while wearing contact lenses. Accordingly, accommodative demand during viewing likely varied across individuals depending on their habitual correction status and residual refractive error. Although neither viewing distance nor residual refractive error was significantly associated with the magnitude of change in distance APCT in the univariable analyses, a potential influence of non-standardized refractive correction and variable accommodative demand cannot be excluded. In addition, CVS-Q scores did not change significantly, possibly reflecting the short exposure duration and/or limited sensitivity of symptom questionnaires to acute changes. Recent reviews have highlighted that studies on digital eye strain have relied predominantly on symptom-based questionnaires, including the CVS-Q, whereas objective assessments of binocular visual function remain limited [9]. Together, these findings indicate that objective measurements of ocular alignment may detect subtle short-term changes that are not accompanied by measurable subjective symptoms.

Prior work by Hirota et al. reported increased fixation instability at very short viewing distances and a higher likelihood of monocular viewing in patients with intermittent exotropia at 20 cm [23], implying that very close smartphone viewing may challenge fixation stability and binocular control. Although the present cohort consisted of non-strabismic adults, the observed baseline-dependent alignment shifts raise the possibility that habitual digital near-work viewing could transiently alter distance alignment in certain individuals, potentially contributing to symptoms in those with limited vergence reserves or borderline binocular control. However, because ocular alignment was not remeasured after smartphone viewing, the recovery time course of these alignment changes remains unknown.

This study has some limitations. First, full refractive correction was not standardized across all participants because the eye tracker precluded spectacle wear and residual refractive errors may have remained in soft-contact-lens wearers. Consequently, refractive status during viewing varied among participants. Second, although smartphone use was limited to social networking, video viewing, and web browsing, viewing distance and content were self-selected rather than being standardized; thus, ecological validity was increased and interindividual differences in visual demands were potentially introduced. Third, the exposure duration was limited to 30 min, and the time course of recovery was not assessed. Fourth, accommodative response, fusional reserves, vergence facility, and other binocular vision metrics were not measured concurrently, which limits mechanistic interpretation. Fifth, pre- and post-viewing APCT measurements were performed by different examiners. Although post-viewing examiners were masked to baseline results, a possible inter-examiner effect cannot be completely excluded. Sixth, a control condition involving sustained near viewing without smartphone use was absent. Therefore, we cannot conclude that the observed changes were specific to smartphone viewing, as the effects of prolonged near-work viewing itself cannot be distinguished. Future studies should incorporate standardized refractive correction, controlled viewing conditions (including viewing distance and content type), a control task or rest condition, and recovery measurements, together with comprehensive accommodative–vergence assessments. Further analyses linking eye-tracking metrics (including gaze-movement velocity and direction) to alignment changes, as well as evaluating the effect of blinking or intermittent distance viewing during smartphone use, may help clarify modifiable factors that influence alignment stability.

## 5. Conclusions

In healthy young adults without manifest strabismus, a small but statistically significant eso-directional shift in ocular alignment at near and distance fixation was observed after 30 min of smartphone viewing. The magnitude of the shift was associated with baseline distance exodeviation. Further studies incorporating appropriate non-smartphone near-viewing control conditions and measurements of accommodative and vergence functions are needed to determine whether these changes are specific to smartphone viewing and to clarify the underlying mechanisms.

## Figures and Tables

**Table 1:** Demographic characteristics of participants at baseline.

Characteristics	Number or Mean (±SD)
Age (years)	23.2 ± 2.5 (range, 20–30)
Sex (male/female)	24:35
SE (D)	
Left eye	−3.36 ± 2.25
Right eye	−3.57 ± 2.4
Ocular deviation (Δ)	
APCT near fixation	−6.2 ± 6.8
APCT distance fixation	−2.0 ± 2.8
SPCT near fixation	−1.6 ± 3.6
SPCT distance fixation	−0.3 ± 1.3
Stereopsis (log arcsec)	
Near	1.6 ± 0.7
Distance	1.7 ± 0.3
Viewing distance (cm)	24.7 ± 4.0

Δ: prism diopters; APCT: alternate prism cover test; D: diopter; SE: spherical equivalent; SPCT: single-prism cover test.

**Table 2:** Multivariable linear regression analysis for predictors of change in APCT at distance deviation after smartphone viewing.

Variable	Estimate (β)	95% CI	*p*-Value
APCT at distance deviation (baseline)	−0.251	−0.374 to −0.128	<0.001
Uncorrected SE in the right eye (baseline)	−0.052	−0.216 to 0.111	0.542
Uncorrected SE interocular difference (baseline)	−0.39	−1.44 to 0.65	0.454

Note: Dependent variable = change in APCT-assessed distance deviation (positive = shift toward esodeviation). No multicollinearity was observed (all VIF < 2). CI: confidence interval; APCT: alternate prism cover test, SE: spherical equivalent.

## Data Availability

Data presented in this study are available on reasonable request from the corresponding author. Data are not publicly available due to privacy and ethical restrictions, as they contain clinical information from human participants.

## Supplementary Materials

The following supporting information can be downloaded at https://www.mdpi.com/article/10.3390/jemr19040086/s1. Figure S1: Distribution of viewing distance during smartphone viewing; Figure S2: (A) Distributions of baseline ocular deviation measured by near APCT, (B) Distributions of baseline ocular deviation measured by distance APCT; Figure S3: (A) Distributions of baseline spherical equivalent refractive error in the left eye, (B) Distributions of baseline spherical equivalent refractive error in the right eye, (C) Distributions of refractive error under smartphone viewing in the left eye, (D) Distributions of refractive error under smartphone viewing in the right eye; Table S1: Mean changes and standardized effect sizes for APCT at distance deviation after smartphone viewing; Table S2: Univariable linear regression analysis for predictors of change in APCT at distance deviation after smartphone viewing; Table S3: Sensitivity analysis using APCT at distance deviation after smartphone viewing as the dependent variable.

## Abbreviations

The following abbreviations are used in this manuscript:

AACE	Acute acquired concomitant esotropia
APCT	Alternate prism cover test
CIs	Confidence intervals
D	Diopter
CVS-Q	Computer Vision Syndrome Questionnaire
^Δ^	Prism diopter
SD	Standard deviation
SPCT	Single-prism cover test
VIF	Variance inflation factor
