# Supplementary material for: Changes in Distance Ocular Deviation After Smartphone Viewing in Young Adults Without Manifest Strabismus
Source: J Eye Mov Res. 2026 Aug 12;19(4):86. doi: 10.3390/jemr19040086 (PMC13514287; doi:10.3390/jemr19040086)
Supplement: Supplementary file 1 [file jemr-19-00086-s001.zip › Supplementary Table S1.pdf]

Outcome	Mean change (Δ)	95% CI	Cohen's dz	95% CI
APCT at near deviation	1.86	0.88–2.84	0.50	0.22–0.76
APCT at distance deviation	0.88	0.50–1.27	0.60	0.32–0.87

Supplementary Table S1. Mean changes and standardized effect sizes for APCT at distance deviation after smartphone viewing. CI: confidence interval; APCT: alternate prism cover test
