# Supplementary material for: Changes in Distance Ocular Deviation After Smartphone Viewing in Young Adults Without Manifest Strabismus
Source: J Eye Mov Res. 2026 Aug 12;19(4):86. doi: 10.3390/jemr19040086 (PMC13514287; doi:10.3390/jemr19040086)
Supplement: Supplementary file 1 [file jemr-19-00086-s001.zip › Supplementary Table S2.pdf]

Variable	β coefficient	95% CI	p-value
APCT at distance deviation (baseline)	−0.267	−0.387 to −0.147	<0.001
Uncorrected SE in the right eye (baseline)	−0.140	−0.299 to 0.018	0.082
Uncorrected SE interocular difference (baseline)	0.560	−0.164 to 1.283	0.127
Horizontal-to-vertical gaze-movement ratio (right eye)	0.6	−0.35 to 1.55	0.211
APCT at near deviation (baseline)	−0.029	−0.086 to 0.028	0.308
Pupil diameter in right diameter	0.217	−0.362 to 0.796	0.456
Change in SE in the right eye	−0.383	−1.674 to 0.908	0.555
SE in the right eye (baseline)	−0.049	−0.285 to 0.187	0.678
Percentage of gaze-movement events with velocities <30°/s (right eye)	0.001	−0.022 to 0.025	0.923
Viewing distance	0.005	−0.094 to 0.103	0.926
SE interocular difference (baseline)	−0.004	−1.293 to 1.284	0.995

Footnote:
Each variable was evaluated separately using univariable linear regression.
Outcome variable = change in APCT-assessed distance deviation after smartphone viewing (positive = shift toward esodeviation). APCT: alternate prism cover test, SE: spherical equivalent

Supplementary Table S2. Univariable linear regression analysis for predictors of change in APCT at distance deviation after smartphone viewing
