# Supplementary material for: Changes in Distance Ocular Deviation After Smartphone Viewing in Young Adults Without Manifest Strabismus
Source: J Eye Mov Res. 2026 Aug 12;19(4):86. doi: 10.3390/jemr19040086 (PMC13514287; doi:10.3390/jemr19040086)
Supplement: Supplementary file 1 [file jemr-19-00086-s001.zip › Supplementary Table S3.pdf]

Predictor	B	SE	Standardized β	95% CI	p-value
APCT at distance deviation (baseline)	0.749	0.061	0.869	0.626 to 0.872	<0.001
Uncorrected spherical equivalent in the right eye (baseline)	-0.052	0.082	-0.052	-0.216 to 0.111	0.524
Uncorrected spherical equivalent interocular difference (baseline)	0.259	0.366	0.057	-0.473 to 0.992	0.481

Footnote:
To address the potential influence of mathematical coupling associated with change-score analysis, an additional multivariable linear regression analysis was performed using APCT-assessed dostance deviation after smartphone viewing as the dependent variable while retaining the same explanatory variables as those in the primary multivariable model (Table 2). B, unstandardized regression coefficient; SE, standard error; CI, confidence interval; APCT, alternate prism cover test.

Supplementary Table S3. Sensitivity analysis using APCT at distance deviation after smartphone viewing as the dependent variable.
